# Testing the causal relationship of fat and sugar intake with depression and cortisol: a Mendelian Randomisation study

**DOI:** 10.1038/s41398-024-03089-2

**Published:** 2024-09-10

**Authors:** Matylda Buczkowska, Eleonora Iob

**Affiliations:** 1 Institute for Global Health, University College London, London, UK; 2 Department of Epidemiology and Public Health, University College London, London, UK; 3 Social, Genetic & Developmental Psychiatry (SGDP) Centre, Institute of Psychiatry, Psychology & Neuroscience, King’s College London, London, UK

**Keywords:** Depression, Genomics

## Abstract

Unhealthy diets high in fat and sugar content may have an impact on psychological health and increase the risk of Major Depressive Disorder (MDD) and stress levels. On the other hand, MDD and stress might be related to food choices and intake. However, it is not clear whether diet, and specifically fat and sugar intake, is causally related to stress and MDD, and whether this relationship may be bi-directional. This study utilised Mendelian Randomisation (MR) to investigate the causal nature of the relationship of fat and sugar intake with MDD and cortisol (as a proxy of stress), and to shed light on the direction of this relationship. Summary-level data for all exposure and outcome variables were obtained from large-scale, non-overlapping GWASs in individuals of European ancestry. Bidirectional analyses were performed: one with macronutrients as exposures and one with MDD/cortisol as exposures. Random-effects inverse-variance weighted regression was used as the primary analytic method for genetic instruments with at least two single nucleotide polymorphisms (SNPs) available (and individual Wald ratio was used when only one SNP was available). Higher levels of genetically predicted relative sugar intake were causally associated with lower MDD risk, for both genome-wide significant p-value threshold of *p* < 1 × 10^−8^, (OR = 0.553, 95% CI: 0.395-0.775) and relaxed p-value threshold of *p* < 1 × 10^−6^ (OR = 0.786, 95% CI: 0.630–0.981). No reverse causality was detected in the opposite direction as MDD was not associated with sugar consumption. The associations observed for all the other pairs of variables were weak and imprecise. A number of limitations was present in the study, such as low-SNP based heritability for some exposures, inability to prove whether variants were correlated with unmeasured confounders and self-reporting of MDD data. Lifestyle and/or pharmacological interventions targeting sugar-related physiological mechanisms may help to reduce depressive symptoms. However, more research is necessary on short- and long-term effects of sugar on the risk of MDD. Additionally, future studies should investigate whether the amount and type of sugar consumed may underlie the impact of sugar on mood and stress levels.

## Introduction

Diet is an important factor for maintenance of good health throughout the life course [1]. Unhealthy dietary habits are associated with an obvious threat to physical well-being and may lead to obesity and other chronic non-communicable conditions [1], but they can also have an impact on psychological health. Hence, patterns of food consumption and their relation to mental health have recently started receiving more attention in research. Nutritional habits have been proposed to play a role in the aetiology and prognosis of Major Depressive Disorder (MDD) [2]. MDD is a major public health problem [3], partially due to a role of socioeconomic status in depression: there is a large body of literature which illustrates the negative association between socioeconomic status and MDD and indicates strategies need to be developed to protect against the development of MDD. Low socioeconomic status has been shown to affect the economic health burden of MDD, with lower access to care, limited insurance and decreased consistency in treatment follow-up of MDD [4]. Although the evidence base is still scarce, previous clinical and observational studies suggest that diet and nutrition may be promising targets for the prevention and treatment of MDD. For example, a meta-analysis of randomised clinical trials found that dietary interventions are potentially contributing to reduction of symptoms of depression across the population [5]. Another review showed that omega-3 fatty and amino acids might have anti-depressant effects [6]. Moreover, balanced diets, such as the Mediterranean diet, have been shown to lower the risk of depressive symptoms, while unhealthy diets characterised by fast foods, snacks and highly palatable “comfort foods” (i.e., tasty, calorically-dense foods containing high amounts of sugars and fats [7]) are linked with higher risk of depressive symptoms [2, 8–11].

However, this association has also been seen in the opposite direction. Negative emotion might be related to food choice and intake that in turn is associated with mood in a bi-directional manner [12]. Some research suggests that depressed individuals show a preference for “comfort foods” as a strategy to alleviate their negative emotions and feelings [12]. One of the most prevalent factors that trigger MDD is stress. Dysregulation of the hypothalamic-pituitary-adrenal (HPA) axis function often leads to hypercortisolism and may be a key biological mechanism underlying the relationship between stress and depression [13]. Cortisol can affect food consumption on the reward basis by increasing food salience and reward feeling [14]. This can happen directly with cortisol influencing the reward pathway through increased opioid and dopamine levels, as well indirectly with cortisol affecting a range of other hormones, such as leptin and insulin, which play a role in regulation of appetite and reward [14]. Higher perceived stress and higher cortisol levels have been be linked with higher fat and sugar intake [1, 12–19], overall higher food intake [15] and greater drive to eat [20]. These results support the possibility of a cortisol-induced preference for “comfort food” and contribute to the significance of the stress-diet link [14].

Despite this evidence, it is not clear whether diet is causally related to stress and MDD, and whether this relationship may be bi-directional. Despite randomised clinical trials being considered the gold standard method for addressing causality, they are also often not practical for assessing real world effects of dietary patterns on mental health as the study designs usually have strict inclusion criteria and low external validity [21]. In contrast, observational prospective studies can assess diet as a whole and investigate a range of effects of nutrition on mental health or stress levels across the population. However, due to the lack of randomisation, several health-related and socioeconomic factors can potentially act as confounders of their association.

Of note, there are many discrepancies in the results of clinical trials and observational studies on diet and mental health. For example, one trial (*N* = 10) did not support the hypothesis that higher fat intake contributes to higher levels of cortisol [22], while another trial (*N* = 18 353) found that omega-3 fats may contribute to higher risk of depressive symptoms [23]. Some observational studies also suggest that consumption of high amounts of sugar and saturated fat is not associated with depressive symptoms [24]. Such discrepancies could be explained by differences in the study samples and assessment methods across studies [9], such as the use of self-reported measures which might not accurately capture differences in diet and mood. Self-reported data might also be affected by the presence of social desirability and recall biases [25, 26].

Mendelian Randomisation (MR) provides an alternative approach from the observational studies and randomised clinical trials. It is a genetically informed method which takes advantage of randomly assigned genetic variation at conception prior to the onset of disease as a proxy of exposure, which reduces the chance of confounding [27]. Genetic variants associated with the exposure of interest are used as instrumental variables for assessing causal relationships with the outcome and vice versa [27]. No research to date has used this approach to test the bidirectional relationships of fat and sugar intake with depression and cortisol (i.e., biomarker of stress). MR offers unique opportunities for unravelling causal links in risk factors of psychiatric disorders, especially compared to more traditional research methods: psychiatric disorders, such as MDD often have a multifactorial origin and behavioural confounders that can affect the risk of the disease [28].

### Aims and hypotheses

The aims of this study were to investigate whether the relationship of fat and sugar intake with MDD and cortisol is causal, and to shed light on the direction of this relationship. We applied two-sample MR to test bidirectional associations of fat and sugar intake with plasma cortisol levels and risk of MDD, using results from published genome-wide association studies (GWAS). The following hypotheses were tested:

- Higher intake of fat and sugar is associated with higher levels of plasma cortisol.
- Higher intake of fat and sugar is associated with an increased risk of MDD.
- The association of fat and sugar intake with plasma cortisol levels and risk of MDD may also be bidirectional, whereby higher levels of plasma cortisol and risk of MDD are associated with higher intake of fat and sugar.

## Materials and methods

### Study design

**Fig. 1:**
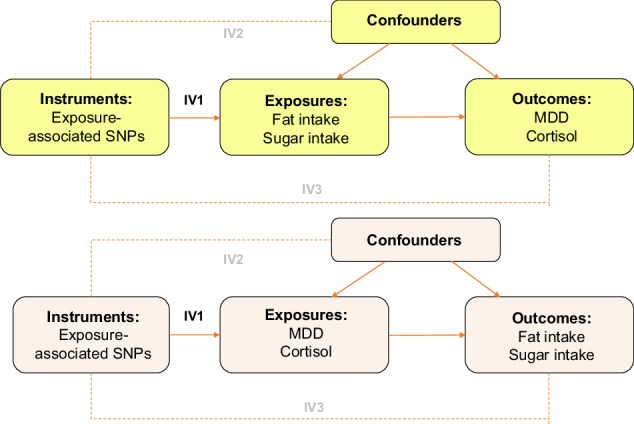
Study design and Mendelian Randomisation (MR) assumptions. Note: Solid arrows represent the pathways which are hypothesised to exist, while dashed arrows represent the pathways which are hypothesised not to exist, based on MR assumptions. SNPs= single nucleotide polymorphisms, MDD=Major Depressive Disorder, IV1, IV2, IV3 = MR assumption 1, 2, 3.

Bidirectional MR analyses were conducted with relative intake of fat and sugar as (i) the exposure, to assess whether they have a causal effect on plasma cortisol levels and risk of MDD, and as (ii) the outcome, to assess whether plasma cortisol levels and risk of MDD have a causal effect on relative intake of fat and sugar (Fig. 1). Summary-level data for all exposure and outcome variables were obtained from large-scale, non-overlapping GWASs in individuals of European ancestry. Relative fat and sugar intake were considered as proxy measures for unhealthy, snack-type diets while plasma cortisol levels were considered as a proxy for biological stress levels.

### GWAS data sources (Table 1)

#### Macronutrients

Summary statistics for relative fat (*N* ~ 264,000) and relative sugar (*N* ~ 231,000) intake were obtained from the European-ancestry GWAS meta-analysis [29], which included a number of cohorts from the UK (UK Biobank, ALSPAC, Fenland), the Netherlands (Lifelines, RSI/II/III), USA (FHS, HRS, GARNET, HIPFX, WHIMS+) and the international consortia EPIC-InterAct and DietGen (DietGen only analysed fat, protein and carbohydrate intake, but not sugar intake). Previous-day (UK Biobank) or habitual (all other cohorts) dietary intake was assessed with comprehensive food-item questionnaires. All cohorts used self-report questionnaires containing ≥70 food items. The relative contributions of fat, protein, carbohydrate, and sugar to total energy intake were calculated, and individuals on calorie- or macronutrient-restricted diets were excluded.

**Table 1:** Overview of the GWAS datasets.

Phenotype	Consortium	Total sample size	SNP-based heritability	Z-value^1^	Genome-wide significant loci	Source
*a) Fat and sugar intake*						
Relative fat intake	ALSPAC (only mothers), DietGen* (=Nurses Health Study + Health Professionals Followup Study + Women’s Genome Health Study, EPIC-InterAct1, EPIC-InterAct2, Fenland, Framingham Heart Study, Health and Retirement Study, LifeLines, Rotterdam Study 1 + 2 + 3, UK Biobank, Women’s Health Initiative GARNET&HIPFX&WHIMS+ (only non-participants of the Dietary Modification Study).	264,181	0.03	10.00	6	27
Relative sugar intake	ALSPAC (only mothers), EPIC-InterAct1, EPIC-InterAct2, Fenland, Framingham Heart Study, Health and Retirement Study, LifeLines, Rotterdam Study 1 + 2 + 3, UK Biobank, Women’s Health Initiative GARNET&HIPFX&WHIMS+ (only non-participants of the Dietary Modification Study).	230,648	0.05	11.75	10	27
*b) Mental health disorders*						
MDD	PGC (UK Biobank and 23andMe datasets were excluded)	143,265	0.09	22.50	44	28
*c) Stress-related biomarkers*						
Plasma cortisol levels	CORNET (CROATIA-Vis, CROATIA-Korcula, CROATIA-Split, ORCADES, Rotterdam Study, NFBC1966, Helsinki Birth Cohort Study 1934–44, ALSPAC (mothers excluded), PREVEND, and PIVUS).	12,597	0.005	0.10	1	29

*GWAS* Genome-wide Association Studies, *ALSPAC* The Avon Longitudinal Study of Parents and Children, *EPIC* European Prospective Investigation into Cancer, *PGC* Psychiatric Genomics Consortium, *CORNET* CORtisol NETwork.

^1^Z-value = SNP-based heritability/Standard Error. The power of a GWAS dataset is likely sufficient if there is at least one genome-wide significant locus, the SNP-based heritability is ≥ 0.05, and the Z-value is ≥4 [34].

#### MDD and plasma cortisol

Summary statistics for diagnoses of MDD (*N* ~ 143,000) were obtained from the Psychiatric Genomics Consortium (PGC) [30]. Meta-analytic results that left out UK Biobank participants for MDD were used, in order to avoid sample overlap between the exposure and outcome data. Participants from 23andMe were also excluded owing to access constraints. Summary statistics for diagnoses of plasma cortisol (*N* ~ 13,000) were obtained from the CORtisol NETwork (CORNET) consortium [31], including a meta-analysis of nine cohorts of European descent. Mean morning plasma cortisol levels in the included studies ranged from 305 to 765 nmol/L. The genetic association estimates were adjusted for age, sex, and genetic principal components.

### Selection of genetic instruments

Two sets of genetic instruments were chosen for each exposure variable: first set included only single nucleotide polymorphisms (SNPs) reported as genome-wide significant (*p* < 1 × 10^−8^), while the other set included SNPs meeting a relaxed p-value threshold (*p* < 5 × 10^−8^). SNPs that were correlated at r^2^ > 0.001 were clumped to make sure that the genetic variants used in the study were independent. When SNPs for the exposure were not available in the summary statistics of the outcome, they were replaced with overlapping proxy SNPs in high-linkage disequilibrium (r^2^ > 0.8). Following that, outcome and exposure data were harmonised, and data pruning was applied to drop potential duplicate summary sets. Steiger directionality and filtering tests were performed to drop SNPs which had a greater effect on the outcome than on the exposure, as this would indicate that the assumption that exposure causes the outcome is not valid.

### Statistical analyses

Bidirectional pathways between macronutrients and MDD/plasma cortisol were assessed by considering analysis in two directions: one with macronutrients as exposures and one with MDD/plasma cortisol as exposures. Random-effects inverse-variance weighted (IVW) regression [32] was used as the primary analytic method for genetic instruments with at least two SNPs available. For genetic instruments involving a single SNP, individual Wald ratios (WR) are presented instead [32]. As measures of effect size, odds ratios (OR) are reported for binary outcomes and standardised beta coefficients (*β*) for continuous outcomes. Sensitivity analyses were run to test the three core assumptions for valid instrumental variables. The three assumptions are: (IV1) the genetic variant should be associated with the exposure, (IV2) the genetic variant should not be associated with confounders, (IV3) the genetic variant should affect the outcome only through the exposure pathway [33]. If instrument SNPs show horizontal pleiotropy, IV2 and IV3 are violated [33]. MR methods used in sensitivity analyses to assess and correct potential violations of key MR assumptions included MR-Egger [34], weighted median, weighted mode, MR-PRESSO, and MR-RAPS [35–37]. Finally, Cochran’s (IVW) and Rucker’s (MR Egger) Q tests were performed to detect heterogeneous causal effects when using meta-analytic methods. Single SNP analysis plots and leave-one-out analysis plots were produced. Single SNP and leave-one-out analyses were only performed for the associations with at least three SNPs available. All statistical analyses were conducted in R (version 4.0.2) using the *TwoSampleMR* package.

## Results

The results of the main (IVW/WR) and sensitivity (weighted median, weighted mode, and RAPS) analyses are presented in Tables 2 and 3. Other sensitivity analyses results are presented in Supplementary Tables 1–4 and Supplementary Figs. 1–6.

**Table 2:** Main and sensitivity analyses results with Major Depressive Disorder (MDD) and plasma cortisol as outcomes and relative fat and sugar intake as exposures.

Outcome	Exposure	MR method	N (SNPs)	b	SE	CI (lower)	CI (upper)	*P*-value	Odds ratio^a^/ Standardised effect^b^	CI (lower)	CI (upper)
***Direction 1: Macronutrients -> MDD/Plasma cortisol (p*** <***10e-8)***											
MDD	Relative fat intake	IVW	5	0.236	0.209	−0.174	0.646	0.260	1.266^a^	0.840	1.908
		Egger		−0.319	0.321	−0.948	0.309	0.393	0.727^a^	0.388	1.362
		Weighted median		−0.018	0.216	−0.442	0.405	0.216	0.982^a^	0.643	1.500
		Weighted mode		−0.032	0.216	−0.455	0.391	0.216	0.969^a^	0.635	1.479
		RAPS		0.240	0.173	−0.100	0.579	0.173	1.271^a^	0.905	1.785
	Relative sugar intake	IVW	9	−**0.592**	**0.172**	−**0.930**	−**0.255**	**<0.001**	**0.553**^**a**^	**0.395**	**0.775**
		Egger		0.738	0.971	−1.165	2.642	0.472	2.093^a^	0.312	14.048
		Weighted median		−**0.659**	**0.218**	−**1.086**	−**0.231**	**0.003**	**0.518**^**a**^	**0.338**	**0.794**
		Weighted mode		−0.670	0.361	−1.377	0.039	0.101	0.512^a^	0.252	1.040
		RAPS		−**0.607**	**0.158**	−**0.916**	−**0.298**	**<0.001**	**0.545**^**a**^	**0.400**	**0.742**
Plasma cortisol	Relative fat intake	Wald ratio	1	−1.125	0.679	−2.565	0.096	0.069	0.291^b^	0.077	1.101
		RAPS		−1.126	0.724	−2.653	0.184	0.088	0.291^b^	0.070	1.202
	Relative sugar intake	IVW	4	0.146	0.328	−0.496	0.788	0.656	1.157^b^	0.609	2.198
		Egger		1.306	2.577	−3.745	6.357	0.663	3.692^b^	0.024	576.57
		Weighted median		0.118	0.354	−0.575	0.812	0.738	1.126^b^	0.563	2.252
		Weighted mode		0.048	0.449	−0.831	0.927	0.922	1.049^b^	0.436	2.527
		RAPS		0.148	0.285	−0.410	0.706	0.602	1.160^b^	0.664	2.026
***Direction 1: Macronutrients -> MDD/Plasma cortisol (p*** < ***10e-6)***											
MDD	Relative fat intake	IVW	35	0.008	0.149	−0.284	0.301	0.955	1.009^a^	0.752	1.352
		Egger		−0.248	0.338	−0.911	0.415	0.469	0.780^a^	0.402	1.515
		Weighted median		−0.009	0.167	−0.337	0.319	0.957	0.991^a^	0.714	1.376
		Weighted mode		0.110	0.200	−0.283	0.503	0.588	1.116^a^	0.753	1.653
		RAPS		0.042	0.101	−0.156	0.240	0.679	1.043^a^	0.855	1.271
	Relative sugar intake	IVW	40	−**0.241**	**0.113**	−**0.462**	−**0.020**	**0.033**	**0.786**^**a**^	**0.630**	**0.981**
		Egger		−0.397	0.397	−1.175	0.380	0.323	0.672^a^	0.309	1.463
		Weighted median		−**0.297**	**0.146**	**−0.584**	−**0.010**	**0.043**	**0.743**^**a**^	**0.558**	**0.990**
		Weighted mode		−0.575	0.357	−1.274	0.125	0.115	0.563^a^	0.280	1.133
		RAPS		−**0.235**	**0.095**	−**0.422**	−**0.048**	**0.014**	**0.790**^**a**^	**0.656**	**0.953**
Plasma cortisol	Relative fat intake	IVW	2	−0.542	0.535	−1.591	0.507	0.311	0.582^b^	0.204	1.660
		RAPS		−0.322	0.565	−1.428	0.785	0.569	0.725^b^	0.240	2.191
	Relative sugar intake	IVW	10	0.037	0.178	−0.310	0.385	0.833	1.038^b^	0.733	1.470
		Egger		0.750	0.870	−0.954	2.454	0.414	2.117^b^	0.385	11.637
		Weighted median		0.050	0.318	−0.573	0.673	0.875	1.051^b^	0.564	1.960
		Weighted mode		0.095	0.428	−0.744	0.934	0.829	1.100^b^	0.475	2.544
		RAPS		0.038	0.233	−0.418	0.494	0.870	1.039^b^	0.658	1.639

*MR* Mendelian Randomisation, *MDD* Major Depressive Disorder, *CI* confidence intervals, *SNPs* single nucleotide polymorphisms, *RAPS* Robust Adjusted Profile Score, *IVW* inverse-variance weighting.

The bold values represent statistically significant values (rows for which *p*-value ≤ 0.05).

**Table 3:** Main and sensitivity analyses results with relative fat and sugar intake as outcomes and Major Depressive Disorder (MDD) and plasma cortisol as exposures (*p* < 10e-8 = main analysis, *p* < 10e-6 = sensitivity analysis).

Outcome	Exposure	MR method	N (snps)	b	SE	CI (lower)	CI (upper)	*P*-value	Standardised effect	CI (lower)	CI (upper)
***Direction 1:*** ***MDD/Plasma cortisol -> Macronutrient*** ***(p*** <***10e-8)***											
Relative fat intake	MDD	IVW	2	−0.133	0.091	−0.311	0.045	0.091	0.875	0.733	1.046
		RAPS		−**0.143**	**0.040**	−**0.222**	−**0.064**	**<0.001**	**0.866**	**0.801**	**0.938**
	Plasma cortisol	Wald ratio	1	0.041	0.033	−0.022	0.105	0.202	1.042	0.978	1.111
		RAPS		0.041	0.034	−0.025	0.109	0.224	1.042	0.975	1.115
Relative sugar intake	MDD	IVW	2	0.060	0.053	−0.045	0.165	0.263	1.062	0.956	1.179
		RAPS		0.061	0.040	−0.018	0.141	0.129	1.063	0.982	1.151
	Plasma cortisol	Wald ratio	1	−0.016	0.035	−0.085	0.052	0.638	0.984	0.919	1.053
		RAPS		−0.016	0.036	−0.087	0.054	0.649	0.984	0.916	1.056
***Direction 2: MDD/Plasma cortisol -> Macronutrients (p*** < ***10e-6)***											
Relative fat intake	MDD	IVW	41	−0.001	0.012	−0.035	0.012	0.330	0.989	0.966	1.012
		Egger		0.045	0.040	−0.033	0.124	0.266	1.046	0.967	1.132
		Weighted median		−0.013	0.014	−0.041	0.014	0.343	0.987	0.960	1.014
		Weighted mode		−0.018	0.026	−0.071	0.034	0.493	0.982	0.932	1.034
		RAPS		−0.012	0.010	−0.032	0.008	0.225	0.988	0.969	1.008
	Plasma cortisol	IVW	6	−0.002	0.011	−0.023	0.019	0.873	0.998	0.977	1.020
		Egger		−0.023	0.016	−0.056	0.009	0.227	0.977	0.946	1.009
		Weighted median		−0.015	0.012	−0.038	0.009	0.224	0.985	0.962	1.009
		Weighted mode		−0.014	0.012	−0.038	0.010	0.302	0.986	0.963	1.010
		RAPS		−0.002	0.010	−0.22	0.019	0.864	0.998	0.978	1.019
Relative sugar intake	MDD	IVW	41	0.004	0.011	−0.018	0.026	0.740	1.004	0.982	1.026
		Egger		−0.004	0.038	−0.078	0.071	0.921	0.996	0.925	1.073
		Weighted median		−0.006	0.015	−0.036	0.023	0.670	0.994	0.965	1.023
		Weighted mode		−0.014	0.034	−0.081	0.054	0.690	0.986	0.922	1.055
		RAPS		0.004	0.011	−0.017	0.025	0.717	1.004	0.983	1.025
	Plasma cortisol	IVW	6	0.016	0.009	−0.002	0.033	0.086	1.015	0.998	1.033
		Egger		0.040	0.017	0.007	0.074	0.079	1.041	1.007	1.077
		Weighted median		0.020	0.013	−0.006	0.046	0.129	1.020	0.994	1.048
		Weighted mode		0.025	0.013	−0.001	0.052	0.118	1.026	0.999	1.053
		RAPS		0.015	0.011	−0.006	0.037	0.166	1.015	0.994	1.038

*MR* Mendelian Randomisation, *MDD* Major Depressive Disorder, *CI* Confidence Intervals, *SNPs* Single Nucleotide Polymorphisms, *RAPS* Robust Adjusted Profile Score, *IVW* inverse-variance weighting.

The bold values represent statistically significant values (rows for which *p*-value ≤ 0.05).

### Main analyses

#### Direction 1: Effects of relative fat and sugar intake on stress levels (measured by levels of plasma cortisol) and risk of MDD

Higher levels of genetically predicted sugar intake were causally associated with lower risk of MDD, for both genome-wide significant p-value threshold (IVW OR = 0.553, 95% CI: 0.395−0.775) and relaxed p-value threshold (IVW OR = 0.786, 95% CI: 0.630−0.981). There was no statistical evidence of a causal association between sugar intake and cortisol (IVW OR = 1.157, 95% CI: 0.609−2.198), as well as between fat intake and MDD (IVW OR = 1.266, 95% CI: 0.840−1.908) and cortisol (WR OR = 0.291, 95% CI: 0.077−1.101) at genome-wide significant p-value threshold. At relaxed p-value threshold, the results were similar between sugar intake and cortisol (IVW OR = 1.038, 95% CI: 0.733−1.470), as well as between fat intake and MDD (IVW OR = 1.009, 95% CI: 0.752−1.352) and cortisol (WR OR = 0.582, 95% CI: 0.204−1.660).

#### Direction 2: Effects of stress levels (measured by levels of plasma cortisol) and MDD on relative fat and sugar intake

There was weak evidence of causal associations in this direction at genome-wide significant p-value threshold between sugar intake and MDD (IVW OR = 1062, 95% CI: 0.956−1.179) and cortisol (IVW OR = 0.984, 95% CI: 0.919−1.053), as well as between fat intake and MDD (IVW OR = 0.875, 95% CI: 0.733−1.046) and cortisol (WR OR = 0.291, 95% CI: 0.978−1.111). Similar results were obtained at relaxed p-value threshold for all association pairs.

### Sensitivity analyses

#### Direction 1: Effects of relative fat and sugar intake on stress levels (measured by levels of plasma cortisol) and risk of MDD

Weighted median and RAPS methods analyses with relative sugar intake as the exposure and risk of MDD as the outcome showed significant effects in the same direction as those observed in the main analyses, with similar confidence intervals (Table 2). The association between relative sugar intake and risk of MDD was imprecise (i.e., the confidence interval of the estimated OR included zero). However, it should be noted that these sensitivity analysis methods would result in lower statistical power than IVW due to stricter assumptions.

MR-PRESSO test showed potential heterogeneity and unbalanced horizontal pleiotropy for the association between relative sugar intake as the exposure and risk of MDD as the outcome was tested for both p-value thresholds (Supplementary Table 4). There was no evidence of heterogeneity when the intercept of MR-Egger was calculated (Supplementary Table 2). The Steiger directionality suggested that the overall direction of the observed MR effects was correct for almost all associations, except for the association between sugar intake and cortisol levels (genome-wide significant p-value threshold) and the association between fat intake and cortisol levels (relaxed p-value threshold). However, both effects were driven by a small number of SNPs, and the directions were significant when using the other available threshold. Single SNP analyses and leave-one-out SNP analyses provided consistent results with the main analysis and no outliers were found (Supplementary Figs. 1−4).

#### Direction 2: Effects of stress levels (measured by levels of plasma cortisol) and MDD on relative fat and sugar intake

RAPS analyses with MDD as the exposure and relative fat intake as the outcome indicated that MDD is associated with lower relative fat intake (RAPS standardised effect=0.866, 95% CI: 0.801, 0.938) when genome-wide p-value threshold was used (Table 3). However, the effect was driven by only one SNP. Additionally, that association was displaying heterogeneity for both IVW and Egger regressions Q statistics (Supplementary Table 1). There were no other precise associations found in this direction using MR-Egger, weighted median, weighted mode or RAPS methods (Table 3).

MR-Egger intercept testing, Q statistics and MR-PRESSO tests highlighted no evidence of heterogeneity or horizontal pleiotropy for the rest of the associations in this direction (Supplementary Tables 1, 2 and 4). The Steiger directionality test suggested that the overall direction of the observed MR effects was correct (Supplementary Table 3). Single SNP analyses and leave-one-out SNP analyses provided consistent results with the main analysis and no outliers were identified (Supplementary Figs. 5 and 6).

## Discussion

In this study, large-scale GWAS datasets were used to test the causal nature and direction of the relationship of sugar and fat intake with MDD and stress. The results showed that higher genetically predicted sugar intake had a causal protective effect on the risk of MDD. Furthermore, no reverse causality was detected, with MDD not being associated with higher sugar consumption. Associations between other exposures and outcomes were weak and imprecise in both directions.

Interestingly, most population-based studies have found an opposite effect, with higher sugar consumption being linked with increased MDD risk. For example, a meta-analysis of 10 observational studies concluded that consumption of sugar-sweetened beverages was associated with an increased risk of depression [38]. Another systematic review, which looked at patients living with type 2 diabetes, found that increased sugar intake in food was associated with depression [39].

There might be a few explanations of the association between sugar and MDD presented in the Results. Firstly, high-sugar foods, such as chocolate, have psychoactive chemicals targeting opioid receptors in the central nervous system, and their low-to-moderate consumption can affect happiness levels [40]. Additionally, glucose has been shown to boost cognitive performance during increasing mental efforts, and might enhance learning and memory in healthy humans, for example through enhancement of neurocognitive markers and medial temporal and frontal activation [41, 42]. Cognitive dysfunction is one of potential mediators of functional impairment in MDD, while lowered ability to concentrate is one of the diagnosis criteria of MDD [43]. Secondly, type of sugar was not differentiated. Existing research has shown that natural fructose from the fruit might be associated with improved cognitive function, and fibre, vitamins and minerals in fruit can counteract the potential negative impacts of sugar [44]. Sugar also affects changes in dopamine signalling and sensitisation of D-1 dopamine and mu-1 opioid receptors. Removal of high-sugar products from diet might lead to depressive behaviours, similar to withdrawal symptoms in drugs of dependence [44].

Moreover, metabolic properties of sugar contribute to stress relief through peripheral (e.g., glucocorticoid receptor signalling in adipose tissue) and brain (e.g., plasticity in brain reward regions) mechanisms [11]. Hence, the relationship between sugar and MDD and its underlying mechanisms deserve further attention in future research.

There was no association between sugar intake and stress levels nor fat intake, MDD risk and stress levels. These results are contradictory to existing research. For instance, an observational study using UK Biobank data found that diets characterised by high consumption of chocolate, confectionery, butter, high-fat cheese and added sugars were linked with higher risk of depression and anxiety [45]. A study done on China Health and Nutrition Survey participants found that perceived levels of stress were associated with a preference for fast foods and sugary drinks [46]. It is possible that the associations between sugar intake and stress levels as well as fat intake and stress levels were not detected in our study due to limited statistical power of the underlying GWAS datasets (e.g., cortisol data).

### Strengths and limitations

The main strength of the study was the application of genetically informed MR analyses which are less prone to confounding bias than traditional observational studies. The datasets used came from the largest non-overlapping genetic consortia and biobanks available. Additionally, for each exposure-outcome pair, sensitivity analyses with a relaxed threshold p-value were conducted and several robust MR methods were used to validate major MR assumptions and allow for various potential patterns of horizontal pleiotropy.

Despite these strengths, a number of limitations should be noted. Firstly, SNP-based heritability was low for some exposures. For instance, genetic variants used in the study only accounted for 0.54% of the variation in morning plasma cortisol, and the overall sample size of the cortisol GWAS was much smaller than that of other datasets used in the study, which may have reduced the statistical power of the analyses involving cortisol as an exposure or outcome and therefore the null findings from the study cannot rule out alternative hypothesis. To address this, we run analyses with a relaxed threshold p-value, which enabled us to use a larger number of SNPs as genetic instruments for cortisol. Although this method has a potential risk of basing the research on false genetic instruments, MR-RAPS was utilised as a sensitivity analysis to address weak instrument bias.

Secondly, it is impossible to prove whether or not the variant is correlated with an unmeasured confounder. There was also a large time gap between conception, when the genotype is determined, and time of exposure and outcome measurement, therefore timing of the causal effects cannot be determined. Therefore, the estimated MR effect should be interpreted as a lifetime association. Another limitation is self-reported dietary data in the UK Biobank. To assess diet, UK Biobank participants were asked to report what foods and drinks they had consumed during the preceding 24 h, by answering questions about the frequency of intake of approximately 200 commonly consumed foods and drinks, which may not provide accurate information about usual dietary habits. Moreover, neither DSM nor ICD were utilised in some of the studies assessing MDD presence in PGC. Non-stringent inclusion criteria and not relying on DSM/ICD-based diagnoses introduces the risk of unwanted population heterogeneity. Lack of standardisation of exposures/outcomes and not applying widely used diagnostic criteria to measure MDD might lead to bias in the results.

The p-values were not adjusted for multiple testing comparisons, therefore there is a higher chance that true null hypotheses were rejected. Additionally, self-reporting often leads to biased results as reporting relies on participants’ memory and willingness to share all the foods eaten, even if excess calories are consumed. Moreover, a number of potential psychosocial factors, such as educational attainment, could be potential confounders in diet, MDD and stress relationship. In the case of same genetic variants being correlated to fat and sugar consumption, as well as those potential confounders, MR assumptions might be violated. Finally, we only analysed data from participants of European descent. Any recommendations and conclusions reached in the study are therefore only applicable to European populations and should not be generalised to individuals of other descents who may have a different genetic make-up.

## Conclusions

In conclusion, this study used genetically informed methods to understand the causal relationship between sugar and fat consumption, MDD risk, and cortisol levels. Our findings suggest that higher sugar consumption may have a protective effect against MDD. Therefore, lifestyle and/or pharmacological interventions targeting sugar-related physiological mechanisms may help to alleviate stress and reduce depressive symptoms. For example, future research may test the effects of sugar-mimicking drugs on the brain as a way to alleviated MDD symptoms and stress levels, which would not cause potentially harmful side effects of high sugar consumption (such as diabetes and obesity). Further research on the amount and type of sugar consumed and its effect on risk of MDD is also necessary. For example, future MR studies can be conducted focusing specifically on added sugar, or selected food items high in sugar content, instead of relative sugar in the diet. Additionally, to further investigate the links between sugar, fat and stress, analyses could be repeated if a GWAS for plasma cortisol with a larger sample size will become available in the future.

## Supplementary information

Supplementary Files

## Data Availability

Summary-level data for the exposures and outcomes were extracted from large-scale GWASs or genetic consortia, including the UK Biobank, the Psychiatric Genomics Consortium and CORNET.

## Code availability

The code of the statistical analyses can be accessed on GitHub: https://github.com/matyldab97/2-sample-MR-study-of-unhealthy-diets-MMR-and-chronic-stress. Supplementary information is available at TP’s website.
