# Supplementary material for: Testing the causal relationship of fat and sugar intake with depression and cortisol: a Mendelian Randomisation study
Source: Transl Psychiatry. 2024 Sep 10;14:368. doi: 10.1038/s41398-024-03089-2 (PMC11387734; doi:10.1038/s41398-024-03089-2)
Supplement: Supplementary file 1 — Supplementary Files [file 41398_2024_3089_MOESM1_ESM.docx]

**SUPPLEMENTARY TABLES AND FIGURES**

**Supplementary Table 1.** Heterogeneity statistics for IVW and Egger regressions.

| ***Direction 1: Macronutrients -> MDD/Plasma cortisol (p<10e-8)*** | | | | | | |
| --- | --- | --- | --- | --- | --- | --- |
| **Outcome** | **Exposure** | **MR method** | **N (SNPs)** | **Q** | **Q_df** | **Q P-value** |
| MDD | Relative fat intake | IVW | 5 | 6.020 | 4 | 0.198 |
|  |  | Egger |  | 1.839 | 3 | 0.607 |
|  | Relative sugar intake | IVW | 9 | 10.297 | 8 | 0.245 |
|  |  | Egger |  | 8.070 | 7 | 0.326 |
| Plasma cortisol | Relative fat intake | IVW | 2 | 0.064 | 1 | 0.801 |
|  | Relative sugar intake | IVW | 4 | 4.111 | 3 | 0.250 |
|  |  | Egger |  | 3.723 | 2 | 0.155 |
| ***Direction 1: Macronutrients -> MDD/Plasma cortisol (p<10e-6)*** | | | | | | |
| MDD | Relative fat intake | IVW | 35 | **74.151** | **34** | **<0.001** |
|  |  | Egger |  | **72.578** | **33** | **<0.001** |
|  | Relative sugar intake | IVW | 40 | **56.826** | **39** | **0.032** |
|  |  | Egger |  | **56.575** | **38** | **0.027** |
| Plasma cortisol | Relative fat intake | IVW | 2 | 0.607 | 1 | 0.436 |
|  | Relative sugar intake | IVW | 10 | 5.599 | 9 | 0.779 |
|  |  | Egger |  | 4.879 | 8 | 0.770 |
| ***Direction 2: MDD/Plasma cortisol -> Macronutrients (p<10e-8)*** | | | | | | |
| Relative fat intake | MDD | IVW | 2 | **6.308** | **1** | **0.012** |
| Relative sugar intake |  | IVW |  | 1.913 | 1 | 0.167 |
| ***Direction 2: MDD/Plasma cortisol -> Macronutrients (p<10e-6)*** | | | | | | |
| Relative fat intake | MDD | IVW | 41 | 58.902 | 40 | 0.027 |
|  |  | Egger |  | **55.779** | **39** | **0.040** |
|  | Plasma cortisol | IVW | 6 | 5.775 | 5 | 0.329 |
|  |  | Egger |  | 2.999 | 4 | 0.558 |
| Relative sugar intake | MDD | IVW | 41 | 47.040 | 40 | 0.206 |
|  |  | Egger |  | 46.989 | 39 | 0.178 |
|  | Plasma cortisol | IVW | 6 | 3.571 | 5 | 0.613 |
|  |  | Egger |  | 0.197 | 4 | >0.99 |
| **Note.** IVW= Inverse-variance weighted (regression), MDD=Major Depressive Disorder, MR=Mendelian Randomisation, SNPs=Single Nucleotide Peptides | | | | | | |

**Supplementary Table 2.** Intercepts of MR-Egger regression.

| ***Direction 1: Macronutrients -> MDD/Plasma cortisol (p<10e-8)*** | | | | |
| --- | --- | --- | --- | --- |
| **Outcome** | **Exposure** | **Egger Intercept** | **SE** | **P-value** |
| MDD | Relative fat intake | 0.018 | 0.006 | 0.404 |
|  | Relative sugar intake | -0.031 | 0.022 | 0.207 |
| Plasma cortisol |  | -0.030 | 0.065 | 0.694 |
| ***Direction 1: Macronutrients -> MDD/Plasma cortisol (p<10e-6)*** | | | | |
| MDD | Relative fat intake | 0.005 | 0.006 | 0.133 |
|  | Relative sugar intake | 0.003 | 0.008 | 0.683 |
| Plasma cortisol |  | -0.015 | 0.017 | 0.421 |
| ***Direction 2: MDD/Plasma cortisol -> Macronutrients (p<10e-6)*** | | | | |
| Relative fat intake | MDD | -0.003 | 0.002 | 0.148 |
|  | Plasma cortisol | 0.005 | 0.003 | 0.171 |
| Relative sugar intake | MDD | 0.001 | 0.002 | 0.838 |
|  | Plasma cortisol | -0.006 | 0.003 | 0.140 |
| **Note.** MDD=Major Depressive Disorder, MR=Mendelian Randomisation, SE=Standard Error | | | | |

**Supplementary Table 3.** Steiger directionality test results.

| ***Direction 1: Macronutrients -> MDD/Plasma cortisol (p<10e-8)*** | | | | | |
| --- | --- | --- | --- | --- | --- |
| **Outcome** | **Exposure** | **R2 Exposure** | **R2 Outcome** | **Correct direction** | **P-value** |
| MDD | Relative fat intake | 0.001 | 5.738e-05 | TRUE | <0.001 |
|  | Relative sugar intake | 0.002 | 1.847e-04 | TRUE | <0.001 |
| Plasma cortisol | Relative fat intake | **3.413e-03** | **3.760e-04** | **FALSE** | **0.920** |
|  | Relative sugar intake | **0.001** | **4.469e-04** | **TRUE** | **0.142** |
| ***Direction 1: Macronutrients -> MDD/Plasma cortisol (p<10e-6)*** | | | | |  |
| MDD | Relative fat intake | 0.001 | 5.738e-05 | TRUE | <0.001 |
|  | Relative sugar intake | 0.005 | 4.635e-04 | TRUE | <0.001 |
| Plasma cortisol | Relative fat intake | **2.943e-03** | **9.766e-05** | **TRUE** | **0.427** |
|  | Relative sugar intake | 0.002 | 4.469e-04 | TRUE | 0.039 |
| ***Direction 2: MDD/Plasma cortisol -> Macronutrients (p<10e-8)*** | | | | |  |
| Relative fat intake | MDD | 0.001 | 7.519e-05 | TRUE | <0.001 |
|  | Plasma cortisol | 0.004 | 6.159e-06 | TRUE | <0.001 |
| Relative sugar intake | MDD | 0.001 | 1.865e-05 | TRUE | <0.001 |
|  | Plasma cortisol | 0.004 | 9.632e-07 | TRUE | <0.001 |
| ***Direction 2: MDD/Plasma cortisol -> Macronutrients (p<10e-6)*** | | | | |  |
| Relative fat intake | MDD | 0.007 | 2.282e-04 | TRUE | <0.001 |
|  | Plasma cortisol | 0.043 | 2.197e-05 | TRUE | <0.001 |
| Relative sugar intake | MDD | 0.007 | 2.045e-04 | TRUE | <0.001 |
|  | Plasma cortisol | 0.043 | 2.462e-05 | TRUE | <0.001 |
| **Note.** MDD=Major Depressive Disorder | | | | | |

**Supplementary Table 4.** MR-PRESSO tests: Global test for horizontal pleiotropy and comparison of raw and outlier-corrected causal estimates.

| ***Direction 1: Macronutrients -> MDD/Plasma cortisol (p<10e-8)*** | | | | | | |
| --- | --- | --- | --- | --- | --- | --- |
| **Outcome** | **Exposure** | **MR Analysis** | **Causal estimate** | **SD** | **T-stat** | **P-value** |
| MDD | Relative fat intake | Raw | 0.236 | 0.209 | 1.127 | 0.323 |
|  | Relative sugar intake | Raw | **-0.592** | **0.172** | **-3.442** | **0.009** |
| Plasma cortisol |  | Raw | 0.146 | 0.328 | 0.445 | 0.686 |
| ***Direction 1: Macronutrients -> MDD/Plasma cortisol (p<10e-6)*** | | | | |  |  |
| MDD | Relative fat intake | Raw | 0.039 | 0.149 | 0.262 | 0.795 |
|  |  | Outlier-corrected | 0.126 | 0.117 | 1.075 | 0.290 |
|  | Relative sugar intake | Raw | **-0.223** | **0.112** | **-2.000** | **0.052** |
|  |  | Outlier-corrected | **-0.270** | **0.101** | **-2.664** | **0.011** |
| Plasma cortisol | Relative sugar intake | Raw | 0.037 | 0.178 | 0.211 | 0.837 |
| ***Direction 2: MDD/Plasma cortisol -> Macronutrients (p<10e-6)*** | | | | |  |  |
| Relative fat intake | MDD | Raw | -0.012 | 0.012 | -0.974 | 0.336 |
|  |  | Outlier-corrected | -0.009 | 0.009 | -0.967 | 0.339 |
|  | Plasma cortisol | Raw | -0.002 | 0.012 | -0.160 | 0.879 |
| Relative sugar intake | MDD | Raw | 0.004 | 0.011 | 0.331 | 0.742 |
|  | Plasma cortisol | Raw | 0.015 | 0.009 | 1.717 | 0.147 |
| **Note.** MDD=Major Depressive Disorder, MR=Mendelian Randomisation, SD=Standard Deviation | | | | | | |


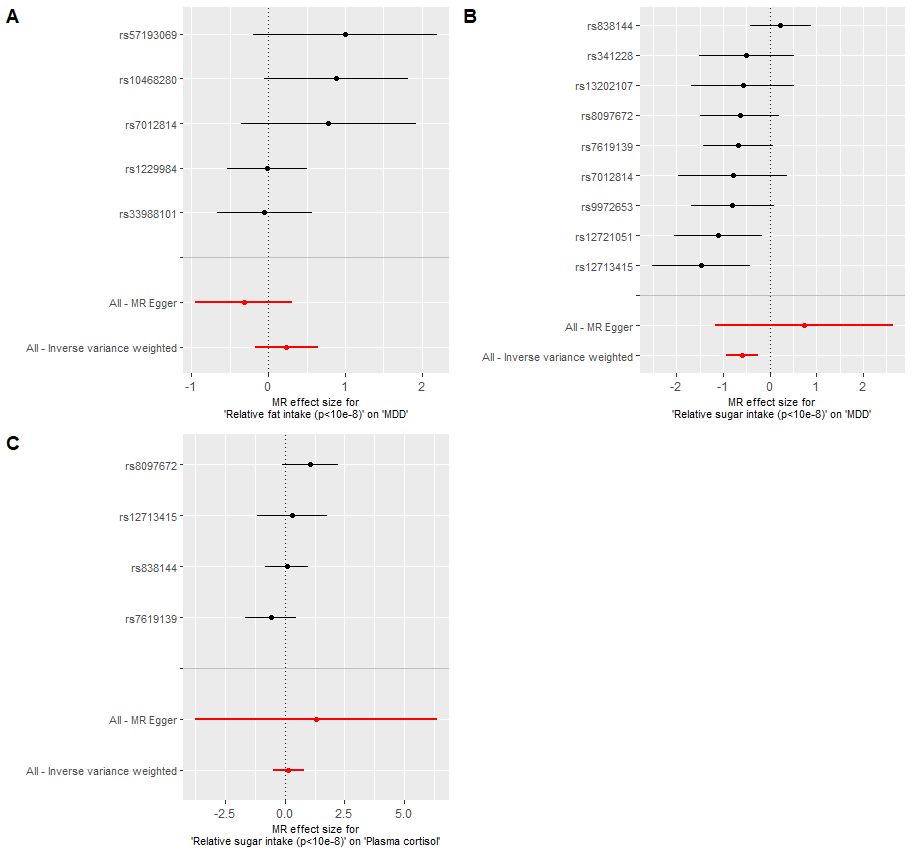


**Supplementary Figure 1.** Single SNP analysis for Direction 1 analyses for genome-wide significant p-value threshold (p<10e-8).

**Note.** MDD=Major Depressive Disorder; MR=Mendelian Randomisation; A=effect of fat intake on risk of MDD, B=effect of sugar intake on risk of MDD, C= effect of sugar intake on plasma cortisol levels


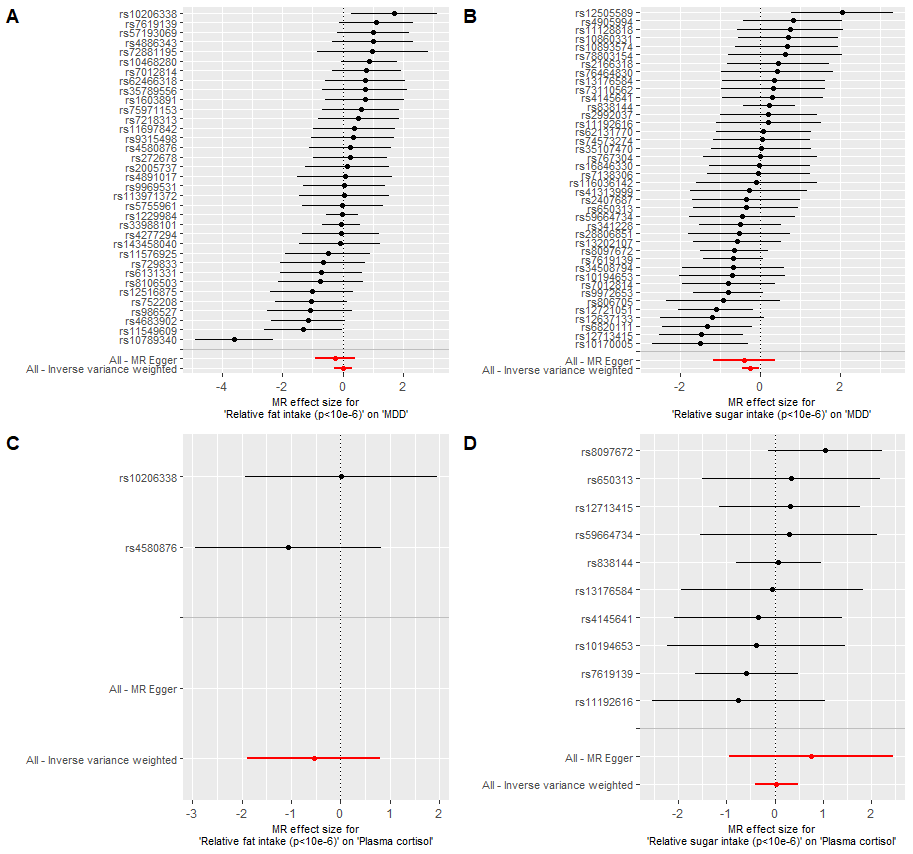


**Supplementary Figure 2.** Single SNP analysis for Direction 1 analyses for relaxed p-value threshold (p<10e-6).

**Note.** MDD=Major Depressive Disorder; MR=Mendelian Randomisation; A=effect of fat intake on risk of MDD, B=effect of sugar intake on risk of MDD, C= effect of fat intake on plasma cortisol levels, D= effect of sugar intake on plasma cortisol levels


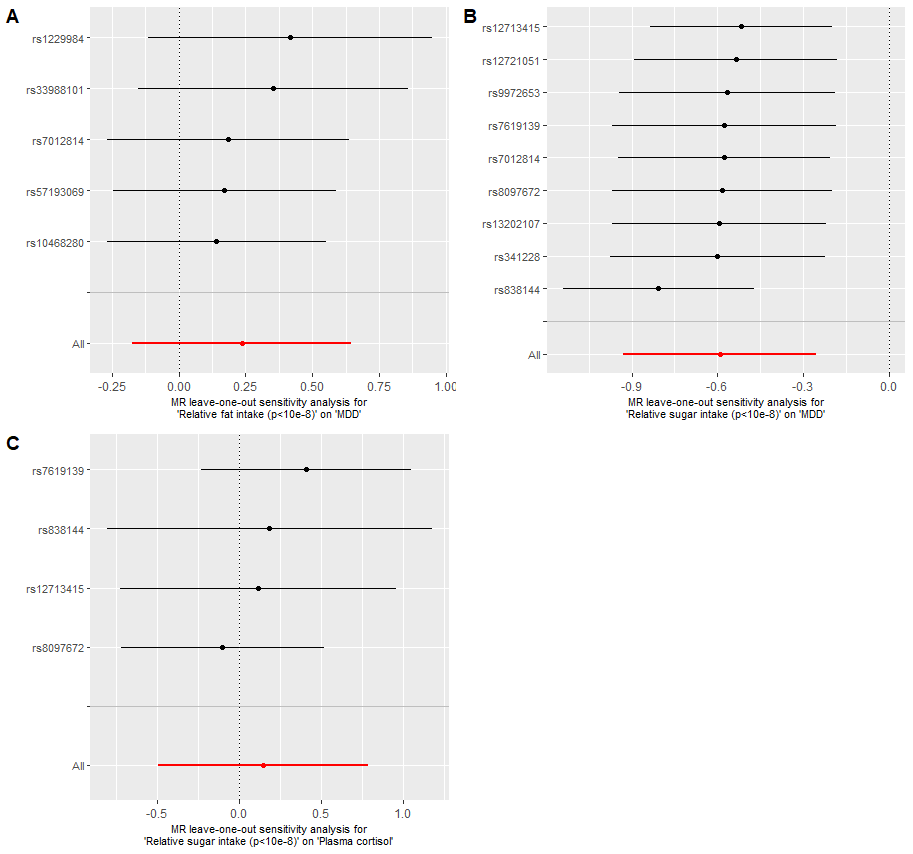


**Supplementary Figure 3.** Leave-one-out SNP analysis for Direction 1 analyses for genome-wide significant p-value threshold (p<10e-8).

**Note.** MDD=Major Depressive Disorder; MR=Mendelian Randomisation; A=effect of fat intake on risk of MDD, B=effect of sugar intake on risk of MDD, C= effect of sugar intake on plasma cortisol levels


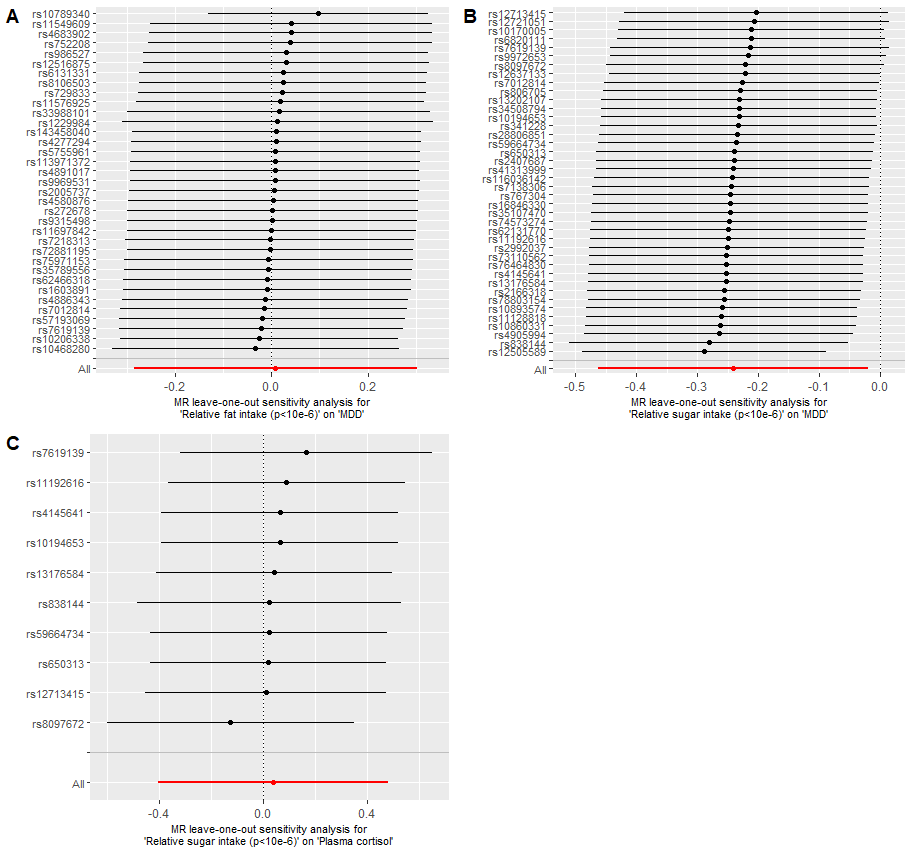


**Supplementary Figure 4.** Leave-one-out SNP analysis for Direction 1 analyses for relaxed p-value threshold (p<10e-6).

**Note.** MDD=Major Depressive Disorder; MR=Mendelian Randomisation; A=effect of fat intake on risk of MDD, B=effect of sugar intake on risk of MDD, C= effect of sugar intake on plasma cortisol levels


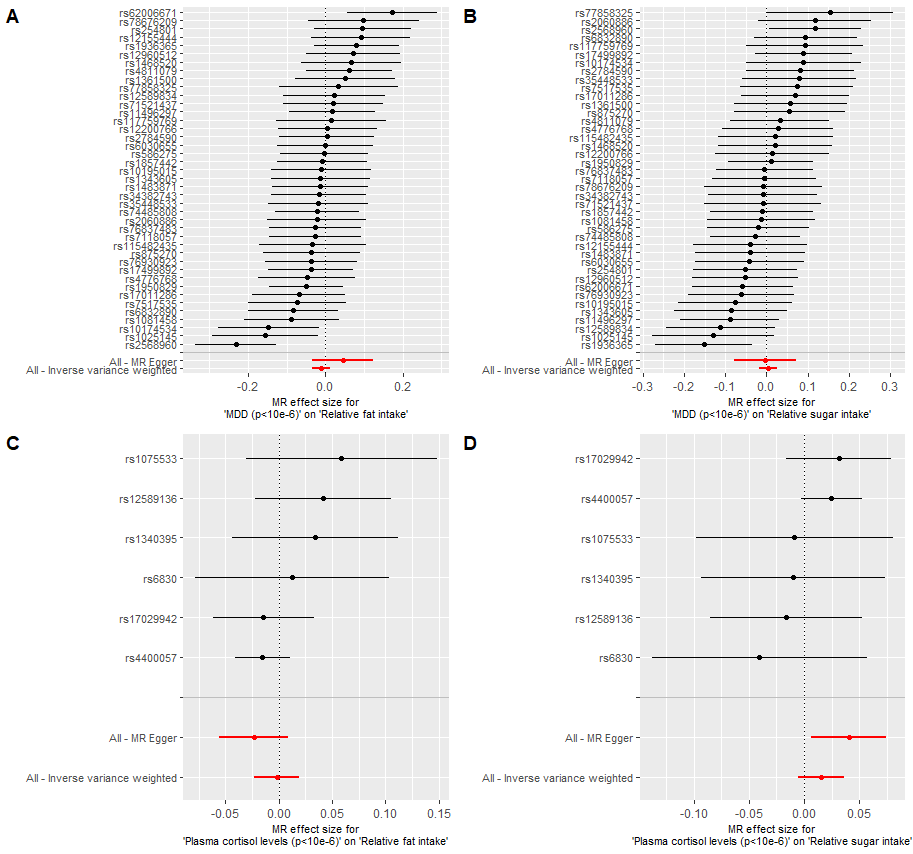


**Supplementary Figure 5.** Single SNP analysis for Direction 2 analyses for relaxed p-value threshold (p<10e-6).

**Note.** MDD=Major Depressive Disorder; MR=Mendelian Randomisation; A=effect of MDD on fat intake, B=effect of MDD on sugar intake, C= effect of plasma cortisol levels on fat intake, D= effect of plasma cortisol levels on sugar intake


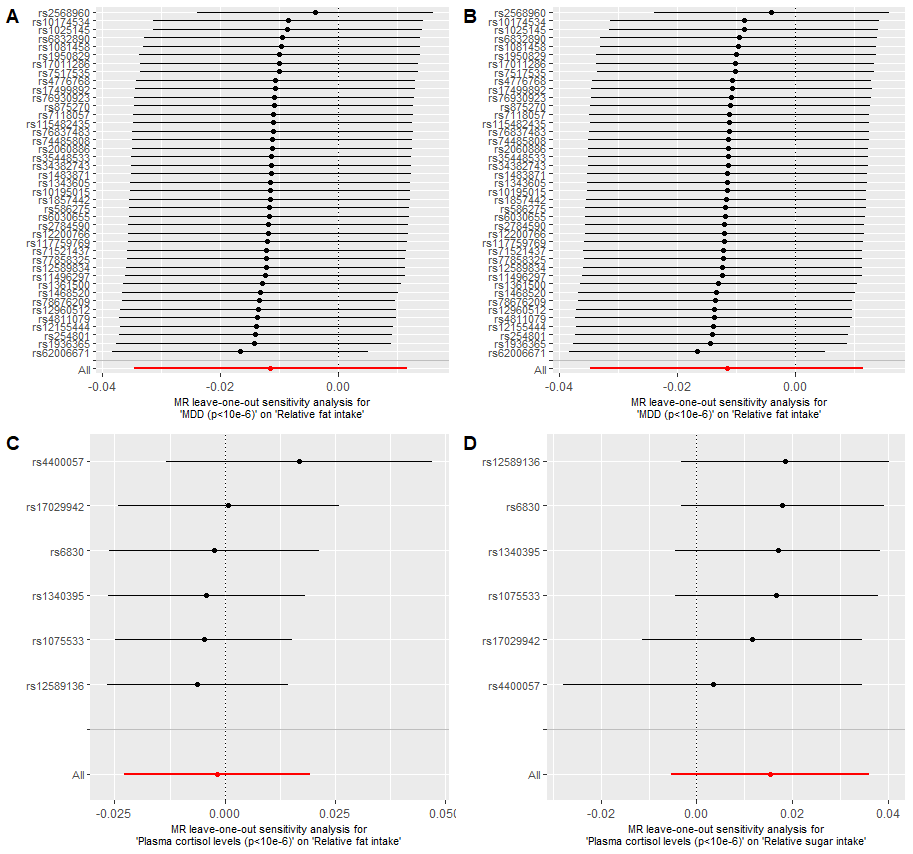


**Supplementary Figure 6.** Leave-one-out analysis for Direction 2 analyses for relaxed p-value threshold (p<10e-6).

**Note.** MDD=Major Depressive Disorder; MR=Mendelian Randomisation; A=effect of MDD on fat intake, B=effect of MDD on sugar intake, C= effect of plasma cortisol levels on fat intake, D= effect of plasma cortisol levels on sugar intake.

**Supplementary Table 5.** SNPs included and excluded from analyses.

| **Outcome** | **Exposure** | **SNP** | **Effect allele exposure** | **Other allele exposure** | **Effect allele outcome** | **Other allele outcome** | **Steiger direction** | **Steiger p-value** |
| --- | --- | --- | --- | --- | --- | --- | --- | --- |
| Sugar | Cortisol | rs12589136* | T | G | T | G | TRUE | 0.000 |
| Sugar | Cortisol | rs1075533 | A | G | A | G | TRUE | 0.001 |
| Sugar | Cortisol | rs1340395 | T | C | T | C | TRUE | 0.000 |
| Sugar | Cortisol | rs17029942 | A | G | A | G | TRUE | 0.000 |
| Sugar | Cortisol | rs4400057 | A | G | A | G | TRUE | 0.000 |
| Sugar | Cortisol | rs6830 | A | G | A | G | TRUE | 0.000 |
| Fat | Cortisol | rs12589136* | T | G | T | G | TRUE | 0.016 |
| Fat | Cortisol | rs1075533 | A | G | A | G | TRUE | 0.000 |
| Fat | Cortisol | rs1340395 | T | C | T | C | TRUE | 0.000 |
| Fat | Cortisol | rs17029942 | A | G | A | G | TRUE | 0.000 |
| Fat | Cortisol | rs4400057 | A | G | A | G | TRUE | 0.000 |
| Fat | Cortisol | rs6830 | A | G | A | G | TRUE | 0.000 |
| Sugar | MDD | rs1950829* | A | G | A | G | TRUE | 0.000 |
| Sugar | MDD | rs2568960* | A | G | A | G | TRUE | 0.001 |
| Sugar | MDD | rs10174534 | A | G | A | G | TRUE | 0.005 |
| Sugar | MDD | rs10195015 | A | G | A | G | TRUE | 0.003 |
| Sugar | MDD | rs1025145 | A | G | A | G | TRUE | 0.004 |
| Sugar | MDD | rs1081458 | A | T | A | T | TRUE | 0.000 |
| Sugar | MDD | rs11496297 | A | G | A | G | TRUE | 0.001 |
| Sugar | MDD | rs115482435 | T | G | T | G | TRUE | 0.000 |
| Sugar | MDD | rs117759769 | A | G | A | G | TRUE | 0.003 |
| Sugar | MDD | rs12155444 | T | G | T | G | TRUE | 0.001 |
| Sugar | MDD | rs12200766 | A | G | A | G | TRUE | 0.000 |
| Sugar | MDD | rs12589834 | A | G | A | G | TRUE | 0.007 |
| Sugar | MDD | rs12960512 | T | C | T | C | TRUE | 0.001 |
| Sugar | MDD | rs1343605 | A | C | A | C | TRUE | 0.004 |
| Sugar | MDD | rs1361500 | T | C | T | C | TRUE | 0.002 |
| Sugar | MDD | rs1468520 | A | G | A | G | TRUE | 0.001 |
| Sugar | MDD | rs1483871 | A | C | A | C | TRUE | 0.001 |
| Sugar | MDD | rs17011286 | A | G | A | G | TRUE | 0.002 |
| Sugar | MDD | rs17499892 | A | C | A | C | TRUE | 0.001 |
| Sugar | MDD | rs1857442 | T | C | T | C | TRUE | 0.000 |
| Sugar | MDD | rs1936365 | C | G | C | G | TRUE | 0.012 |
| Sugar | MDD | rs2060886 | T | C | T | C | TRUE | 0.009 |
| Sugar | MDD | rs254801 | A | C | A | C | TRUE | 0.000 |
| Sugar | MDD | rs2784590 | T | C | T | C | TRUE | 0.002 |
| Sugar | MDD | rs34382743 | T | C | T | C | TRUE | 0.000 |
| Sugar | MDD | rs35448533 | T | C | T | C | TRUE | 0.004 |
| Sugar | MDD | rs4776768 | T | C | T | C | TRUE | 0.001 |
| Sugar | MDD | rs4811079 | C | G | C | G | TRUE | 0.000 |
| Sugar | MDD | rs586275 | A | G | A | G | TRUE | 0.000 |
| Sugar | MDD | rs6030655 | T | C | T | C | TRUE | 0.001 |
| Sugar | MDD | rs62006671 | A | G | A | G | TRUE | 0.001 |
| Sugar | MDD | rs6832890 | C | G | C | G | TRUE | 0.002 |
| Sugar | MDD | rs7118057 | A | G | A | G | TRUE | 0.000 |
| Sugar | MDD | rs71521437 | C | G | C | G | TRUE | 0.000 |
| Sugar | MDD | rs74485808 | A | G | A | G | TRUE | 0.001 |
| Sugar | MDD | rs7517535 | A | G | A | G | TRUE | 0.002 |
| Sugar | MDD | rs76837483 | T | G | T | G | TRUE | 0.000 |
| Sugar | MDD | rs76930923 | A | G | A | G | TRUE | 0.002 |
| Sugar | MDD | rs77858325 | T | C | T | C | TRUE | 0.014 |
| Sugar | MDD | rs78676209 | C | G | C | G | TRUE | 0.000 |
| Sugar | MDD | rs875270 | T | C | T | C | TRUE | 0.001 |
| Fat | MDD | rs1950829* | A | G | A | G | TRUE | 0.000 |
| Fat | MDD | rs2568960* | A | G | A | G | TRUE | 0.041 |
| Fat | MDD | rs10174534 | A | G | A | G | TRUE | 0.017 |
| Fat | MDD | rs10195015 | A | G | A | G | TRUE | 0.000 |
| Fat | MDD | rs1025145 | A | G | A | G | TRUE | 0.007 |
| Fat | MDD | rs1081458 | A | T | A | T | TRUE | 0.003 |
| Fat | MDD | rs11496297 | A | G | A | G | TRUE | 0.000 |
| Fat | MDD | rs115482435 | T | G | T | G | TRUE | 0.000 |
| Fat | MDD | rs117759769 | A | G | A | G | TRUE | 0.000 |
| Fat | MDD | rs12155444 | T | G | T | G | TRUE | 0.004 |
| Fat | MDD | rs12200766 | A | G | A | G | TRUE | 0.000 |
| Fat | MDD | rs12589834 | A | G | A | G | TRUE | 0.000 |
| Fat | MDD | rs12960512 | T | C | T | C | TRUE | 0.002 |
| Fat | MDD | rs1343605 | A | C | A | C | TRUE | 0.000 |
| Fat | MDD | rs1361500 | T | C | T | C | TRUE | 0.001 |
| Fat | MDD | rs1468520 | A | G | A | G | TRUE | 0.002 |
| Fat | MDD | rs1483871 | A | C | A | C | TRUE | 0.000 |
| Fat | MDD | rs17011286 | A | G | A | G | TRUE | 0.001 |
| Fat | MDD | rs17499892 | A | C | A | C | TRUE | 0.000 |
| Fat | MDD | rs1857442 | T | C | T | C | TRUE | 0.000 |
| Fat | MDD | rs1936365 | C | G | C | G | TRUE | 0.001 |
| Fat | MDD | rs2060886 | T | C | T | C | TRUE | 0.000 |
| Fat | MDD | rs254801 | A | C | A | C | TRUE | 0.001 |
| Fat | MDD | rs2784590 | T | C | T | C | TRUE | 0.000 |
| Fat | MDD | rs34382743 | T | C | T | C | TRUE | 0.000 |
| Fat | MDD | rs35448533 | T | C | T | C | TRUE | 0.000 |
| Fat | MDD | rs4776768 | T | C | T | C | TRUE | 0.001 |
| Fat | MDD | rs4811079 | C | G | C | G | TRUE | 0.000 |
| Fat | MDD | rs586275 | A | G | A | G | TRUE | 0.000 |
| Fat | MDD | rs6030655 | T | C | T | C | TRUE | 0.000 |
| Fat | MDD | rs62006671 | A | G | A | G | TRUE | 0.017 |
| Fat | MDD | rs6832890 | C | G | C | G | TRUE | 0.001 |
| Fat | MDD | rs7118057 | A | G | A | G | TRUE | 0.000 |
| Fat | MDD | rs71521437 | C | G | C | G | TRUE | 0.000 |
| Fat | MDD | rs74485808 | A | G | A | G | TRUE | 0.000 |
| Fat | MDD | rs7517535 | A | G | A | G | TRUE | 0.002 |
| Fat | MDD | rs76837483 | T | G | T | G | TRUE | 0.000 |
| Fat | MDD | rs76930923 | A | G | A | G | TRUE | 0.001 |
| Fat | MDD | rs77858325 | T | C | T | C | TRUE | 0.000 |
| Fat | MDD | rs78676209 | C | G | C | G | TRUE | 0.002 |
| Fat | MDD | rs875270 | T | C | T | C | TRUE | 0.001 |
| MDD | Sugar | rs12713415* | C | G | C | G | TRUE | 0.154 |
| MDD | Sugar | rs12721051* | C | G | C | G | TRUE | 0.026 |
| MDD | Sugar | rs13202107* | A | G | A | G | TRUE | 0.008 |
| MDD | Sugar | rs341228* | T | C | T | C | TRUE | 0.004 |
| MDD | Sugar | rs7012814* | A | G | A | G | TRUE | 0.006 |
| MDD | Sugar | rs7619139* | A | T | A | T | TRUE | 0.000 |
| MDD | Sugar | rs8097672* | A | T | A | T | TRUE | 0.002 |
| MDD | Sugar | rs838144* | T | C | T | C | TRUE | 0.000 |
| MDD | Sugar | rs9972653* | T | G | T | G | TRUE | 0.006 |
| MDD | Sugar | rs10170005 | T | C | T | C | TRUE | 0.244 |
| MDD | Sugar | rs10194653 | A | T | A | T | TRUE | 0.042 |
| MDD | Sugar | rs10860331 | T | G | T | G | TRUE | 0.035 |
| MDD | Sugar | rs10893574 | T | C | T | C | TRUE | 0.038 |
| MDD | Sugar | rs1104608 | C | G | C | G | TRUE | 0.010 |
| MDD | Sugar | rs11128818 | T | C | T | C | TRUE | 0.050 |
| MDD | Sugar | rs11192616 | T | C | T | C | TRUE | 0.008 |
| MDD | Sugar | rs116036142 | T | C | T | C | TRUE | 0.006 |
| MDD | Sugar | rs12505589 | T | C | T | C | TRUE | 0.653 |
| MDD | Sugar | rs12637133 | A | G | A | G | TRUE | 0.137 |
| MDD | Sugar | rs13176584 | A | C | A | C | TRUE | 0.011 |
| MDD | Sugar | rs16846330 | T | C | T | C | TRUE | 0.001 |
| MDD | Sugar | rs2166318 | A | G | A | G | TRUE | 0.021 |
| MDD | Sugar | rs2407687 | A | G | A | G | TRUE | 0.017 |
| MDD | Sugar | rs28806851 | A | G | A | G | TRUE | 0.023 |
| MDD | Sugar | rs2992037 | A | G | A | G | TRUE | 0.006 |
| MDD | Sugar | rs34508794 | C | G | C | G | TRUE | 0.036 |
| MDD | Sugar | rs35107470 | A | G | A | G | TRUE | 0.003 |
| MDD | Sugar | rs41313999 | A | G | A | G | TRUE | 0.010 |
| MDD | Sugar | rs4145641 | A | C | A | C | TRUE | 0.010 |
| MDD | Sugar | rs4905994 | T | C | T | C | TRUE | 0.036 |
| MDD | Sugar | rs59664734 | A | G | A | G | TRUE | 0.021 |
| MDD | Sugar | rs62131770 | T | C | T | C | TRUE | 0.003 |
| MDD | Sugar | rs650313 | A | G | A | G | TRUE | 0.017 |
| MDD | Sugar | rs6820111 | A | G | A | G | TRUE | 0.144 |
| MDD | Sugar | rs7138306 | A | G | A | G | TRUE | 0.005 |
| MDD | Sugar | rs73110562 | A | G | A | G | TRUE | 0.015 |
| MDD | Sugar | rs74573274 | T | C | T | C | TRUE | 0.002 |
| MDD | Sugar | rs76464830 | A | T | A | T | TRUE | 0.017 |
| MDD | Sugar | rs767304 | A | G | A | G | TRUE | 0.002 |
| MDD | Sugar | rs78803154 | A | G | A | G | TRUE | 0.034 |
| MDD | Sugar | rs806705 | T | C | T | C | TRUE | 0.071 |
| MDD | Fat | rs10468280* | A | G | A | G | TRUE | 0.011 |
| MDD | Fat | rs1229984* | T | C | T | C | TRUE | 0.000 |
| MDD | Fat | rs33988101* | T | G | T | G | TRUE | 0.000 |
| MDD | Fat | rs57193069* | A | G | A | G | TRUE | 0.048 |
| MDD | Fat | rs7012814* | A | G | A | G | TRUE | 0.004 |
| MDD | Fat | rs10206338 | A | G | A | G | TRUE | 0.423 |
| MDD | Fat | rs10789340 | A | G | A | G | FALSE | 0.152 |
| MDD | Fat | rs113971372 | A | G | A | G | TRUE | 0.007 |
| MDD | Fat | rs11549609 | T | C | T | C | TRUE | 0.282 |
| MDD | Fat | rs11576925 | C | G | C | G | TRUE | 0.033 |
| MDD | Fat | rs11697842 | A | G | A | G | TRUE | 0.021 |
| MDD | Fat | rs12516875 | A | G | A | G | TRUE | 0.112 |
| MDD | Fat | rs143458040 | T | G | T | G | TRUE | 0.007 |
| MDD | Fat | rs1603891 | A | G | A | G | TRUE | 0.049 |
| MDD | Fat | rs2005737 | A | G | A | G | TRUE | 0.009 |
| MDD | Fat | rs272678 | T | G | T | G | TRUE | 0.005 |
| MDD | Fat | rs35789556 | A | G | A | G | TRUE | 0.045 |
| MDD | Fat | rs4277294 | A | T | A | T | TRUE | 0.003 |
| MDD | Fat | rs4580876 | A | G | A | G | TRUE | 0.012 |
| MDD | Fat | rs4683902 | A | C | A | C | TRUE | 0.224 |
| MDD | Fat | rs4886343 | A | G | A | G | TRUE | 0.098 |
| MDD | Fat | rs4891017 | A | G | A | G | TRUE | 0.008 |
| MDD | Fat | rs5755961 | T | G | T | G | TRUE | 0.006 |
| MDD | Fat | rs6131331 | A | C | A | C | TRUE | 0.043 |
| MDD | Fat | rs62466318 | T | C | T | C | TRUE | 0.046 |
| MDD | Fat | rs7218313 | A | C | A | C | TRUE | 0.027 |
| MDD | Fat | rs72881195 | A | G | A | G | TRUE | 0.066 |
| MDD | Fat | rs729833 | T | C | T | C | TRUE | 0.049 |
| MDD | Fat | rs752208 | A | G | A | G | TRUE | 0.109 |
| MDD | Fat | rs75971153 | T | C | T | C | TRUE | 0.027 |
| MDD | Fat | rs7619139 | A | T | A | T | TRUE | 0.090 |
| MDD | Fat | rs8106503 | T | C | T | C | TRUE | 0.053 |
| MDD | Fat | rs873756 | C | G | C | G | TRUE | 0.184 |
| MDD | Fat | rs9315498 | A | C | A | C | TRUE | 0.017 |
| MDD | Fat | rs986527 | A | G | A | G | TRUE | 0.137 |
| MDD | Fat | rs9969531 | C | G | C | G | TRUE | 0.006 |
| Cortisol | Sugar | rs12713415* | C | G | C | G | TRUE | 0.359 |
| Cortisol | Sugar | rs7619139* | A | T | A | T | TRUE | 0.426 |
| Cortisol | Sugar | rs8097672* | A | T | A | T | FALSE | 0.933 |
| Cortisol | Sugar | rs838144* | T | C | T | C | TRUE | 0.051 |
| Cortisol | Sugar | rs10194653 | A | T | A | T | TRUE | 0.511 |
| Cortisol | Sugar | rs11192616 | T | C | T | C | TRUE | 0.793 |
| Cortisol | Sugar | rs13176584 | A | C | A | C | TRUE | 0.303 |
| Cortisol | Sugar | rs4145641 | A | C | A | C | TRUE | 0.470 |
| Cortisol | Sugar | rs59664734 | A | G | A | G | TRUE | 0.454 |
| Cortisol | Sugar | rs650313 | A | G | A | G | TRUE | 0.495 |
| Cortisol | Fat | rs10206338* | A | G | A | G | TRUE | 0.297 |
| Cortisol | Fat | rs873756 | C | G | C | G | TRUE | 0.310 |
| * signifies that the SNP met the p-value threshold of 10^-8  SNP=Single Nucleotide Polymorphism  MDD=Multiple Depressive Disorder | | | | | | | | |
